# Frontier on Alzheimer’s Disease

**DOI:** 10.3390/ijms24097748

**Published:** 2023-04-24

**Authors:** Carlo Cervellati, Giovanni Zuliani

**Affiliations:** Department of Translational Medicine and for Romagna, University of Ferrara, Via Luigi Borsari 46, 44121 Ferrara, Italy

Although substantial progress has been made in the last two decades, there are still important unfilled gaps in the understanding of the pathomechanism of Alzheimer’s disease (AD). Any attempts to simplistically describe the molecular processes underlying the development of this widely spread neurodegenerative disorder have miserably failed. Initially, it was postulated that the aggregation of β-amyloid (Aβ) peptide in senile plaques was the primary and central event in AD onset and progression [1]. Years of widespread basic and clinical research has progressively added new pieces to the puzzle. The current, although not unique, view is that it is a disease which may result from various combinations of a number of abnormalities, besides Aβ, in primis, protein Tau (primer of neurofibrillary tangles), neuroinflammation, mitochondrial dysfunction (leading to oxidative stress), autophagy dysfunction, and brain hypoperfusion [2,3].

The great complexity of AD pathogenesis and pathophysiology has made the research on preventive and disease-modifying therapies highly challenging. The four reviews and two research articles published in this Special Issue cover a wide range of topics but deeply share the same goal: to provide the rationale for new therapeutic strategies for AD.

In the review by Sehar et al. [4], the importance of Aβ as a target of future anti-AD therapies is emphasized. This work summarizes the recent advances in the knowledge of the interaction of Aβ with synapses, mitochondria, microglia, mitochondrial disfunction astrocytes, and tau proteins.

Despite its well-recognized role in AD, many attempts to reduce the Aβ burden have failed to result in significant clinical outcomes. The authors listed the possible drawbacks that may have contributed to the failures of drugs targeting these aberrant peptides. One of the most interesting hypotheses in this regard is the possibility that an excessive inhibition of Aβ formation could have a harmful effect on brain function, due to the suggested role of these peptides in this function (especially memory elaboration).

As suggested by the results reported in Suzuki et al.’s research article [5], the generation of Aβ may not be a good target in the most common form of AD, i.e., late onset AD (LOAD, also referred as sporadic AD), but may be a good target in the inherited familial subtype form (FAD). FAD accounts for approximately 13% of early onset AD (thus, from 1 to 6% of all AD cases). It is caused by mutations in three genes: Aβ precursor protein (APP), a primary substrate of amyloidogenic and non-amyloidogenic pathway, and presenilin 1 and 2 (PSEN1 and PSEN2, respectively), essential components of γ-secretase. This secretase is central in the amyloidogenic pathway, since it catalyzes the cleavage of the β-carboxyl terminal fragment (β-CTF), which is one of the two products of β-secretase action on APP, to form Aβ. Due to its importance, γ-secretase has been tested as a possible drug target for AD. The authors of the published article found that one mutation in APP, namely T714I, significantly decreased the amount of cleavage, and identified secondary APP mutations that restored the cleavage of APP. Moreover, some mutations were found to influence Aβ generation when introduced into mammalian cells.

As highlighted by Merighi et al.’s review [6], neuroinflammation is now regarded as a key component of the pathogenesis and a typical, even if not specific, physiopathological hallmark of AD. Microglia are a primary trigger of this process in the brain. The presence of reactive microglia in AD-vulnerable brain regions (often physically associated with Aβ plaques), even before clinical manifestation of the disease, has been extensively reported. This review covers the most solid evidence linking microglia over-expression and activity (expressed in the release of pro-inflammatory mediators) with AD-related neurodegeneration. These highly specialized brain macrophages act as a “double-edged sword” in this disease. Under physiological conditions, they prevent the formation of neurotoxic Aβ oligomers and tau hyperphosphorylation. On the contrary, upon chronic activation, as occurs in AD, they proactively contribute to disease progression. The authors also stressed the importance of improving the knowledge of the molecular and cellular mechanisms underlying the microglia network, since this hopefully will allow to develop effective anti-AD treatments.

An alternative target for AD treatment could be brain cholesterol. As underpinned in Campos-Peña’s review, cumulating evidence suggests that AD is characterized by a significant alteration of cholesterol metabolism in the brain and also in the periphery [7]. Indeed, changes in the levels of this lipid have direct effects on Aβ formation [8]. Indeed, this reflects the physical-chemical integrity of neuron membranes and other cells in the central nervous system (CNS). In turn, modifications of the membrane lipid composition influence the processing of transmembrane APP and the activity of α- and γ-secretase (as they are also embedded in the neuronal membrane). The cholinergic function, which appears to be impaired already in the early phase of AD, is also related to the lipid homeostasis of the membranes. In particular, multiple reports suggest that cholesterol is able to modulate the function of the nicotinic acetylcholine receptor and alteration of the level of this lipid in membranes negatively affects its ability to interact with neurotransmitters.

Deeper exploration of the (still not completely understood) pathogenesis of AD may be the key to designing novel therapeutic approaches. With this aim, Gil et al. investigated the progression of nuclear alterations in granular neurons of the dental gyrus, which is a complex structure of the human hippocampus, during three different stages of AD: early, intermediate, and late [9]. The researchers evaluated chromatin markers, the components of nuclear lamin; epigenetic histone modifications related to chromatin condensation; and autophagy in post-mortem brain tissues. The collected findings suggest that the nuclear pathology in cells of the dental gyrus is critical in AD from an early stage of the disease. Moreover, the clinical progression of the disease is characterized by an increasing genome dysfunction, finally leading to cognitive decline.

The aforementioned articles clearly demonstrate the high complexity of AD. Rao et al.’s review stresses this concept and provides solid literature evidence supporting the need for a personalized, multi-therapeutic approach to fighting this disease [10]. It is undeniable that none of the mono-therapeutic drugs tested to date have been proven to significantly delay or reverse AD. Some recent clinical trials have demonstrated a decrease in disease progression (with transient attenuation of symptoms), but none have shown a real improvement in the cognitive trajectory of patients. In the authors’ view, the major reason behind these failures lies in the fact that any drug addressing a single abnormality (Aβ, tau, or neuroinflammation) cannot be successful due to the multifactorial and multifaceted nature of AD. This neurodegenerative disease is characterized by a multitude of abnormalities affecting the brain, but also the periphery. The authors described some of these physiopathological conditions, such as insulin resistance, metabolic syndrome, chronic inflammation, hypovitaminosis D, hormonal deficiencies, hyperhomocysteinemia, exposure to metals and chemicals, and infections [11,12]. A multi-therapeutic program (based on improvement in quality of sleep and diet, physical and mental exercise, etc.) that simultaneously approaches these factors and is optimized according to each individual’s genetics may be a promising effective approach to AD treatment.
